# Whole-Genome Sequencing of a Family with Hereditary Pulmonary Alveolar Proteinosis Identifies a Rare Structural Variant Involving *CSF2RA*/*CRLF2*/*IL3RA* Gene Disruption

**DOI:** 10.1038/srep43469

**Published:** 2017-02-24

**Authors:** Chih-Yung Chiu, Shih-Chi Su, Wen-Lang Fan, Shen-Hao Lai, Ming-Han Tsai, Shih-Hsiang Chen, Kin-Sun Wong, Wen-Hung Chung

**Affiliations:** 1 Department of Pediatrics, Chang Gung Memorial Hospital at Keelung, College of Medicine, Chang Gung University, Taoyuan, Taiwan; 2 Division of Pediatric Pulmonology, Chang Gung Memorial Hospital, College of Medicine, Chang Gung University, Taoyuan, Taiwan; 3 Chang Gung Immunology Consortium, Chang Gung Memorial Hospital and Chang Gung University, Taoyuan, Taiwan; 4 Department of Dermatology, Drug Hypersensitivity Clinical and Research Center, Chang Gung Memorial Hospital, Taipei, Linkou and Keelung, Taiwan; 5 Whole-Genome Research Core Laboratory of Human Diseases, Chang Gung Memorial Hospital, Keelung, Taiwan; 6 Division of Pediatric Hematology/Oncology, Chang Gung Memorial Hospital, College of Medicine, Chang Gung University, Taoyuan, Taiwan

## Abstract

Pulmonary alveolar proteinosis (PAP) is a rare pulmonary disease in which the abnormalities in alveolar surfactant accumulation are caused by impairments of GM-CSF pathway attributing to defects in a variety of genes. However, hereditary PAP is extremely uncommon and a detailed understanding in the genetic inheritance of PAP in a family may provide timely diagnosis, treatment and proper intervention including genetic consultation. Here, we described a comprehensive analysis of genome and gene expression for a family containing one affected child with a diagnosis of PAP and two other healthy siblings. Family-based whole-genome analysis revealed a homozygous deletion that disrupts *CSF2RA, CRLF2*, and *IL3RA* gene in the pseudoautosomal region of the X chromosome in the affected child and one of asymptomatic siblings. Further functional pathway analysis of differentially expressed genes in IL-1β-treated peripheral blood mononuclear cells highlighted the insufficiency of immune response in the child with PAP, especially the protection against bacterial infection. Collectively, our results reveal a novel allele as the genetic determinant of a family with PAP and provide insights into variable expressivity and incomplete penetrance of this rare disease, which will be helpful for proper genetic consultation and prompt treatment to avoid mortality and morbidity.

Pulmonary alveolar proteinosis (PAP) is a rare disease characterized by the accumulation of surfactant in the alveoli with a variable clinical course, ranging from an asymptomatic clinical presentation to severely affected respiratory failure^1^,^2^. Surfactant is normally cleared by alveolar macrophages and granulocyte-macrophage colony-stimulating factor (GM-CSF) is crucial for this process. Disruption of GM-CSF signaling by GM-CSF receptor mutations (hereditary form) or by GM-CSF autoantibodies (autoimmune form) or secondary to an underlying clinical condition (secondary form) in humans results in PAP^3^,^4^. Several causes of PAP lead to diverse clinical course, therapy and outcome. A detailed understanding of the genetics and pathogenesis of PAP will likely provide much needed clinical insights.

In clinics, most pediatric cases of PAP can be attributed to defects in a variety of genes involved in surfactant metabolism^5^. Mutations in the genes for surfactant protein deficiency including *SFTPB* (surfactant protein-B), *SFTPC* (surfactant protein-C), *ABCA3* (ATP binding cassette subfamily A member 3)^6^, and *NKX2-1* (thyroid transcription factor 1)^7^ lead to PAP with diffuse lung disease. PAP can also be caused by mutations in genes encoding the GM-CSF receptor (*CSF2RA* and *CSF2RB*)^8^,^9^,^10^. However, a comprehensive analysis of disease-causing alleles and gene expression profile in a family with PAP is still lacking.

Whole-genome sequencing (WGS) is increasingly applied in clinical settings and has been shown to reliably identify rare genetic variants^11^. Family-based genome sequencing enables the identification of the pathogenic alleles in Mendelian disorders^12^. Hereditary PAP is reported to be caused by a variety of genetic aberrations in a recessive inheritance pattern, and these causative mutations vary in different levels of penetrance or even could also be found in asymptomatic family members. WGS provides the most comprehensive collection of an individual’s genetic variation, which could possibly explain for the difference in PAP susceptibility.

The aim of this study was to investigate the genetic basis of PAP in a family by whole-genome sequencing. Family-based analyses of disease-causing variants were conducted. Genetic variant related to PAP was validated and its functional relevance in response to inflammatory stimuli was also assessed.

## Results

### Patients with PAP

**Figure 1:**
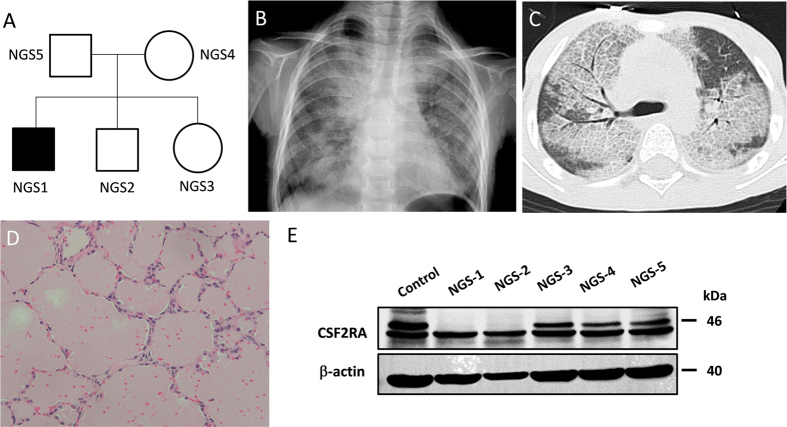
Genetic pedigrees, chest radiograph, high-resolution computed tomography (HRCT) scan of the chest, and lung histopathology in the child with pulmonary alveolar proteinosis. (**A**) Pedigree of family with cases of pulmonary alveolar proteinosis. (**B**) Chest radiograph at presentation showing bilateral diffuse alveolar infiltration with an air bronchogram. (**C**) Chest HRCT showing areas of ground-glass opacification with thickened inter- and intralobular septae, alternated with areas of normal lung. (**D**) Histological examination showing alveoli filled with granular lipoproteinaceous material, staining pink with periodic acid-Schiff (PAS) stain. (**E**) Protein expression analysis of GM-CSF receptors showing the absence of GM-CSF α chain in NGS1 and NGS2. Cropped blots were shown for clarity. Full-length blots/gels are presented in Supplementary Fig. S1A.

The parents of the family in this study were of indigenous Taiwanese descent and lived in a remote area. This area is rural, not densely populated to have the quality of being descended from the same ancestor as another person. However, there was no evidence of any consanguineous marriage during the three generations. They are healthy and denied any congenital anomaly, or deaths of infants and young children among their relatives. At the time of this study, there were two boys and one girl in this family, at age of 6, 4.5 and 2 respectively (Fig. 1A). The second-born boy and third-born girl are in good condition and have not reported any respiratory symptom.

The first-born boy, born at term, was healthy and asymptomatic in the first 3 years of life. At the age of 3 years, he presented with a 4-month history of malaise, progressive shortness of breath with cough and failure to thrive. Clinical examination and blood tests including the complete blood count, leukocyte differential count and C-reactive protein tests revealed no abnormality except inspiratory crackles in bilateral lung fields. A chest radiograph revealed extensive, bilateral alveolar infiltration with opacities (Fig. 1B). A chest HRCT revealed bilateral extensive ground-glass opacities with interlobular septal thickening (Fig. 1C). Bronchoalveolar lavage (BAL) was performed and revealed cloudy, milk-like fluid. Further BAL DNA analyses have revealed *Pneumocystis jiroveci* infection. An immunodeficiency workup revealed normal immunoglobulin levels and normal B and T cell function. *Pneumocystis jiroveci* infection was treated immediately with intravenous co-trimoxazole (20 mg/kg/d of trimethoprim in 4 divided doses given for 4 weeks), and oral steroids (2 mg/kg/d of prednisolone equivalent) used as adjunctive initial therapy but without remission of symptoms. A thoracoscopic lung biopsy revealed alveoli filled with extensive deposition of granular eosinophilic material, staining with periodic acid Schiff (PAS), consistent with a diagnosis of pulmonary alveolar proteinosis (Fig. 1D).

Therapeutic whole lung lavage was carried out under general anesthesia using double lumen endobronchial tube. A total of about 15 liters (1-liter aliquots) of warm normal saline was instilled into the non-ventilated lung. Chest physiotherapy with the aid of postural positioning was performed for the drainage of proteinaceous effluent. Bilateral whole lung lavage received successful, sustained but not long standing remission. Sequential therapeutic lung lavage using flexible bronchoscopy was carried out approximately every year during follow-up period. *Pneumocystis* was not detected in the following bronchoalveolar lavage aspirates. Initial analyses of protein expressions in GM-CSF receptor revealed that GM-CSF receptor α chain (Abcam, Cambridge, MA, USA) was undetectable not only in the index patient, but also in the patient’s younger brother (Fig. 1E).

### Analysis of Whole-Genome Sequencing

**Figure 2:**
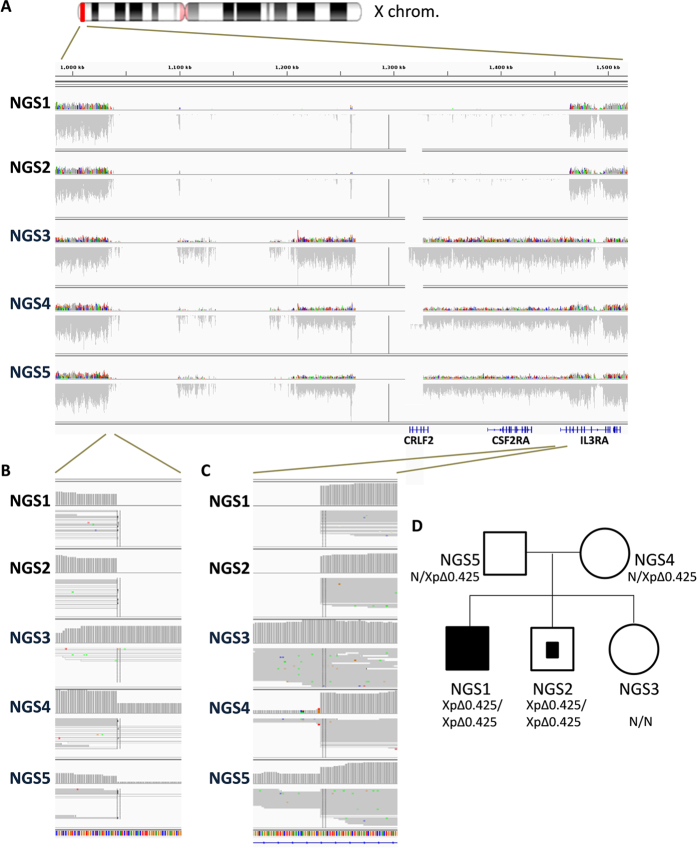
Identification of a structural variant in a family with PAP by whole-genome sequencing. (**A**) Alignment of a subregion of the autosomal region of X chromosome (marked in red) by IGV (Intergative Genomics Viewer) illustrates a deletion of 0.425 Mb (XpΔ0.425) that disrupts the first two exons of the *IL3RA* gene and entire *CRLF2* and *CSF2RA* gene. (**B–C**) Magnified images of the regions surrounding both breakpoints demonstrate the genotype of each family member. (**D**) Pedigree of the patient with PAP in this study. Filled symbol represents the proband, and the partially filled symbol indicates the asymptomatic, homozygous carrier.

To characterize the disease-causal allele for this PAP family, we conducted whole-genome sequencing on all five family members. In our initial attempt to identify single-nucleotide variation (SNV) and small insertion/deletion (INDEL) that inactivate (truncated) the gene product in an autosomal recessive inheritance pattern, no candidate allele was found in *CSF2RA* (GM-CSF receptor α) or in genes for surfactant metabolism such as *SFTPB, SFTPC, ABCA3*, and *NKX2-1* (Supplementary Table S1 and Supplementary Fig. S2). While extending our search onto structural variants and copy-loss variations, a homozygous deletion of 425 kb that encompasses the first two exons of the *IL3RA* gene and entire *CRLF2* and *CSF2RA* gene in the pseudoautosomal region of the X chromosome was detected in the proband and his asymptomatic younger brother (Fig. 2), which is consistent with their abnormal expression of GM-CSF receptor α protein. Both parents were heterozygous for this allele, and the asymptomatic younger sister was normal for this structural variation.

### *CSF2RA, CRLF2,* and *IL3RA* Gene Expression in PBMCs

**Figure 3:**
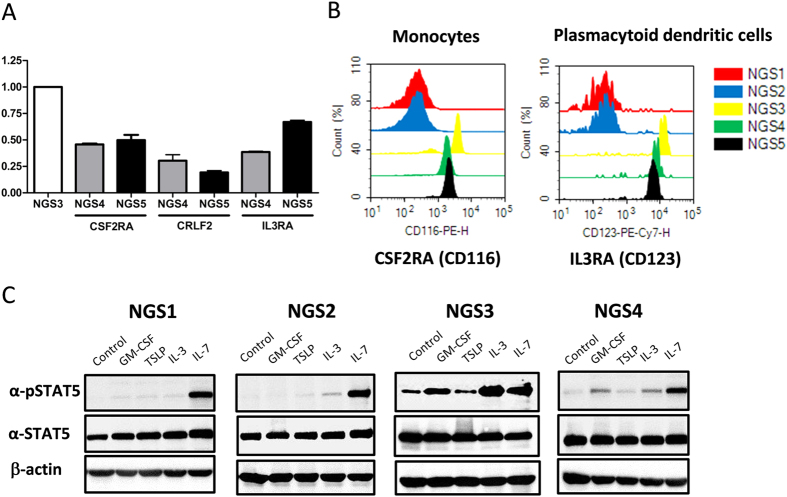
Quantitative real-time PCR analysis, flow cytometry and signaling pathway analysis of *CSF2RA, CRLF2*, and *IL3RA* gene expression. (**A**) Quantitative RT-PCR revealed a decrease in expression of these genes in NGS4 and NGS5 (parents) compared to NGS3 (healthy girl). (**B**) Histograms showed the shift in the fluorescence intensity that was seen for the monocytes (PE-labeled anti-human CSF2RA antibody) and for the plasmacytoid dendritic cells (PE-labeled anti-human IL3RA antibody) in NGS3, NGS4 and NGS5. (**C**) PBMCs stimulated with recombinant human GM-CSF and IL-3 but not TSLP revealed the phosphorylation of STAT5 in NGS3 and NGS4. Extremely low STAT5 phosphorylation was detected in PBMCs with GM-CSF, TSLP and IL-3 stimulation in NGS1 and NGS2. Negative control was cell incubated with PBS without stimulant individually. Cropped blots were shown for clarity. Full-length blots/gels are presented in Supplementary Fig. S1B.

To validate the finding of genome sequencing, gene expression of *CSF2RA, CRLF2*, and *IL3RA* was analysed using RT-PCR. Agarose gel electrophoresis revealed a single target band of the real-time PCR product of these three genes but was not detected in the two boys (NGS1 and NGS2) of the family (Supplementary Fig. S3). Real-Time PCR quantification analysis revealed that these gene expression levels were significantly lower in the parents in comparison with that of the healthy girl (NGS3) (Fig. 3A). Moreover, expression of CSF2RA, CRLF2, and IL3RA protein on PMBCs was assessed using flow cytometry. CSF2RA and IL3RA expression was predominantly observed in monocytes and plasmacytoid dendritic cells, respectively. Consistent with mRNA data, there was a gene-dosage effect for CSF2RA and IL3RA among family members (Fig. 3B). Of note, cell-surface expression of CRLF2 was not detectable on any cell type of peripheral blood cells evaluated.

### Analysis of *CSF2RA, CRLF2*, and *IL3RA* Signaling

JAK-STAT signaling pathway was known to be triggered by GM-CSF binding with GM-CSF receptor α chain (*CSF2RA*), TSLP binding with cytokine receptor-like factor 2 (*CRLF2*), and IL-3 binding with IL-3 receptor α chain (*IL3RA*). STAT5 phosphorylation was therefore used to evaluate the function of *CSF2RA, CRLF2*, and *IL3RA* signaling pathway. The signal of STAT5 phosphorylation in PBMCs triggered by the stimulation with recombinant human GM-CSF and IL-3 was extremely low in the two boys (NGS1 and NGS2) compared to the healthy girl (NGS3) of the family. Furthermore, an approximately 50% decrease in STAT5 phosphorylation was found in the mother (NGS4) in comparison with the healthy girl (NGS3) (Fig. 3C). However, no obvious phosphorylation of STAT5 was detected in PBMCs with TSLP stimulation in all subjects after subtracting the background of their own non-stimulated negative control.

### Differential Gene Expression in Response to IL-1β

**Figure 4:**
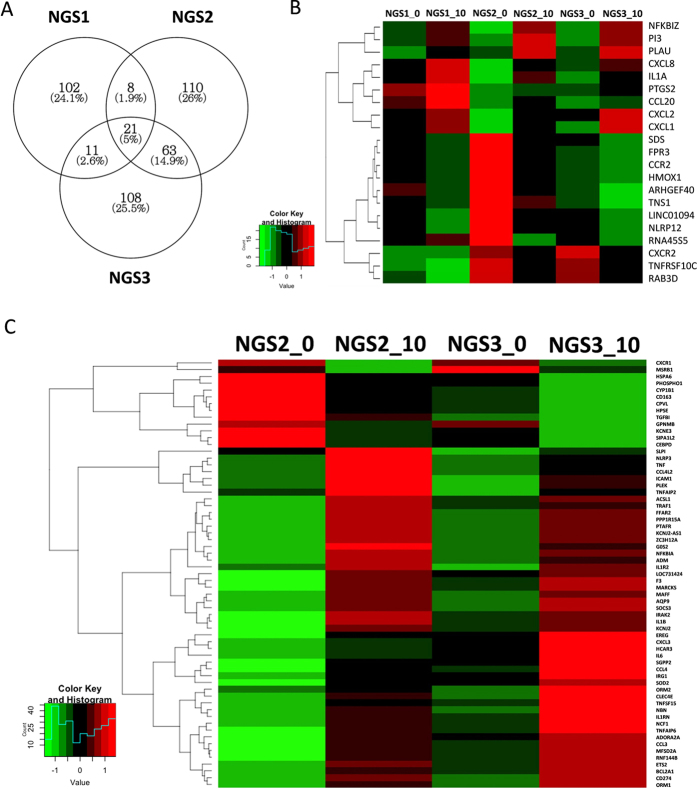
Analysis of gene expression in PMBCs stimulated with IL-1β (10 ng/ml) using RNA sequencing. (**A**) Venn diagram showed the distribution of the genes identified in NGS1, NGS2 and NGS3 (fold change ≥ 2, *P* value < 0.05). (**B**) A heat map of Hierarchical Clustering of 21 genes identified in common in all three subjects. (**C**) A heat map of Hierarchical Clustering of 63 genes identified just in common in NGS2 and NGS3 rather than NGS1. Red or green colours indicate higher and lower expression levels respectively of the genes correlating into a smaller number of uncorrelated variables called principal components.

To further dissect the phenotypic differences among these family members, gene expression analysis of IL-1β-treated PBMCs was performed in three siblings (NGS1, NGS2 and NGS3) to possibly test their ability to react against acute inflammatory processes. 423 differentially expressed transcripts after IL-1β stimulation were identified. Venn diagram showed the distribution of the genes identified in the three paired samples. 21 genes were identified in all three subjects, and 63 genes were concurrently identified in NGS2 and NGS3 but not in NGS1 (Fig. 4A). Differentially expressed genes and heat maps of these 21 and 63 genes clustered using Hierarchical Clustering are shown in Fig. 4B and C respectively. The enrichment of Gene Ontology (GO) in these differentially expressed genes is shown in Supplementary Table S2. Enriched biologic terms being related to inflammatory and defence responses in biological process (BP) were significantly found in the 21 genes identified in common, indicating successful induction of inflammation by IL-1β. In addition to the inflammatory and defence responses in BP, eight significant genes including *IL-6, TNF, ADM, SOCS3, NFKBIA, IL-1β, IRG1* and *PTAFR* related to response to bacterium were found in the 63 genes.

## Discussion

Pulmonary alveolar proteinosis (PAP) is an accumulation of surfactant in the alveoli that is medicated by disruption of GM-CSF signaling^13^. In this study, family-based genome analysis of five members of a family with PAP identified a recessive pattern of homozygous deletion that disrupts *CSF2RA, CRLF2*, and *IL3RA* gene in the pseudoautosomal region of the X chromosome. A similar pattern of transcriptional activation in response to inflammation between the asymptomatic boy and healthy girl may particularly explain the incomplete penetrance of this rare disease. Furthermore, inflammatory defence response, especially in response to bacterium appears to be insufficient in child who developed hereditary PAP, which may cause life-threatening infections.

An imbalance of the production and clearance of surfactant caused by mutations in a variety of genes results in the accumulation of proteinoceous material in the alveoli, leading to the development of PAP^5^. Several studies have identified gene mutations involved in surfactant metabolism using DNA exon-specific amplification and Sanger sequencing for deletions or mutations^8^,^9^,^10^. However, these methods will be infeasible for detecting the pathogenic alleles or genes that encoding extremely large proteins or spanning an immense genomic region. In contrast, whole-genome sequencing provides a more comprehensive approach by read-depth counting, discordant paired-end mappings, and loss of heterozygosity for identifying the genetic causes of disease^14^. In this study, a homozygous large deletion spanning the first two exons of the *IL3RA* gene and entire *CRLF2* and *CSF2RA* gene was identified from comprehensive whole-genome structural variation detection in a family with PAP.

Family-based genome analysis enabled us to narrow the candidate genes for Mendelian disorders. In this Taiwanese family, three genes (*CSF2RA, CRLF2*, and *IL3RA*) are homozygously deleted in the pseudoautosomal region of the human X chromosome, concordant with that PAP is a rare genetic disorder inherited at a recessive pattern. Hereditary PAP caused by various homozygous or compound heterozygous *CSF2RA* mutations has been reported^8^,^10^. To our knowledge, this is the first report of PAP using whole-genome sequencing to define that recessive deletions of large segments of the chromosome involving *CSF2RA* gene contribute to the clinical phenotype.

GM-CSF is a crucial regulator for surfactant homeostasis, and primary PAP occurs when GM-CSF signaling is disrupted^10^,^13^. GM-CSF activates JAK-STAT signaling pathway to induce multiple phagocytic functions of alveolar macrophage and catabolize surfactant properly^15^. In addition to GM-CSF, ligation of TSLP and IL-3 with their cognate receptors, the gene products of *CRLF2* and *IL3RA*, respectively, also activate intracellular STAT5 signaling pathways^16^,^17^. Of note, surface expression and signal transduction of *CRLF2* were undetectable in our investigation, consistent with a previous observation that *CRLF2* gene expression appeared to be detected at only low levels and may not expressed in PBMCs^18^. Despite this, this study has revealed the defects of GM-CSF and IL-3 receptor α chain and their STAT5 phosphorylation signal on blood cells, providing a molecular explanation for the child developing PAP in this study.

The onset of clinical disease of PAP is insidious, with a subacute, symptom-free period ranging from months to several years^2^. The loss of GM-CSF signaling, the crucial pathway for surfactant metabolism, can be compensated by a variety of pro-inflammatory cytokines^19^. Signaling dynamics are also often greatly influenced by positive feedforward and negative feedback loops^20^. In this study, the phenotypic differences in disease severity between both affected siblings with the same gene deletion raise the idea that GM-CSF-dependent signaling and cellular functions may be compensated through cross talks and feedforward loops of abundance of related pathways. This could be particularly explained by the similarity in gene expression profiles between the asymptomatic child with gene deletions and his healthy sister in RNAseq analyses in response to inflammatory stimuli. The penetrance of hereditary PAP caused by the defects in the GM-CSF receptor genes is high but incomplete, highlighting a usefulness of genetic analysis of the proband and other family members for providing proper intervention and genetic consultation.

The overall prognosis for hereditary PAP treated by whole lung lavage is excellent^21^. Initially immunosuppressive therapy because of the interstitial lung parenchymal infiltrates may increase pulmonary opportunistic infections mainly mediated by *Aspergillus* species, *Nocardia* species, *Mycobacterium* species and *Pneumocystis carinii*^22^,^23^,^24^,^25^. However, this susceptibility is multifactorial and may be due to impaired macrophage and neutrophil function^26^,^27^. In this study, transcriptomic and functional analysis revealed impairments of inflammatory, defence and immune responses in the child who developed PAP. A significant inability to response with bacterium was also determined, indicating that opportunistic infections could be common and important causes of morbidity and mortality in patients with PAP.

Limitations of high-throughput DNA sequencing technologies include high error rates, enrichment of rare variants, and a large proportion of missing values^28^,^29^. However, whole-genome sequencing has the potential to identify the structural and noncoding variants typically inaccessible for exome sequencing. A significant strength of this study lies in the family-based WGS, enabling inheritance pattern analyses that permit the identification of precise locations of causative variants.

In conclusion, family-based whole-genome sequencing analysis identified a homozygous deletion that disrupts *CSF2RA, CRLF2*, and *IL3RA* gene in the pseudoautosomal region of the X chromosome as the genetic determinants of a Taiwanese family with PAP. Transcriptomic gene-network and functional pathway analysis highlight the importance of impaired inflammatory response, especially in response to bacterium in the child who developed PAP. Clinically, in children with PAP presenting with persistent disease despite aggressive management, opportunistic infections should be considered, and early identification of the causative pathogen is crucial for reducing morbidity and mortality in such instances.

## Methods

### Participants

A family with cases of pulmonary alveolar proteinosis (PAP) was enrolled. The child with PAP underwent a complete history and examination, and medical records including chest radiographs, high-resolution computed tomography (HRCT) of the chest, surgical lung biopsy and therapeutic bronchoscopy were reviewed. DNA from all family members were subjected to whole-genome sequencing, and comparative analyses were carried out to determine the causative genetic variants. This study was approved by the Institutional Review Board of Chang Gung Memorial Hospital (No. 102-3909B). All experiments in this study were performed in accordance with the relevant guidelines and regulations and written informed consent was obtained from the parents of all study subjects.

### Western Blotting

For Western blots, whole-cell protein extracts were prepared from peripheral blood mononuclear cells (PBMCs) by using radio immunoprecipitation assay (RIPA) lysis buffer (Thermo Scientific, Rockford, USA). Protein concentrations were determined using the BCA Protein Assay Kit (Pierce, Thermo Scientific, Rockford, USA). An equal amount of 30 μg of protein for each sample was separated by electrophoresis on 12% sodium dodecyl sulfate polyacrylamide gels and transferred to nitrocellulose membranes using the iBlot™ DryBlotting device and iBlot™ Transfer stacks. The blots were incubated with the rabbit monoclonal anti-GM-CSF receptor alpha primary antibody at 1:1,000 (Abcam, Cambridge, MA, USA) overnight at 4 °C. Labelling of the first antibodies was detected using relevant secondary antibodies conjugated to horseradish peroxidase (HRP) (1:10,000; Merck Millipore) and detected using a chemiluminescent HRP substrate kit (Immobilon Western; Merck Millipore). The protein bands were detected and scanned by a gel image system (Top Bio Co., MultiGel-21).

### Whole-Genome Sequencing

Blood samples were collected and PBMCs were isolated by centrifugation at 2,000 rpm for 15 min (Eppendorf centrifuge 5810 R) in Ficoll density medium (GE Healthcare). Genomic DNA was extracted by using the DNeasy Blood & Tissue Kit (Qiagen) according to the manufacturer’s instructions and loaded on a 1% agarose gel for quality control. Whole-genome sequencing was performed on five family members. DNA libraries with an insert size of 500–600 bp were prepared according to the protocol provided by Illumina and sequenced on a set of Illumina HiSeq X instruments (Novogene) with paired reads of 150 bp.

### Detection of Genetic Variants

Cleaned sequence data were aligned and mapped to the reference genome (hg19) by Burrows-Wheeler aligner (BWA) using default options^30^. The Genome Analysis Tool Kit (GATK)^31^ was used for detecting SNV and INDEL. The resulting variants were annotated with the ANNOVAR software^32^. To detect copy-number alterations (CNVs), we applied Control-FREEC^33^ to the read count profiles of sequence data. Analysis of structural variants (SVs) was performed by CREST^34^ using the default parameters. Candidate breakpoints in tumors that also appeared in the paired normal specimens were removed before the final SV detection step was performed. Candidate SVs were called by the following filtering criteria: (1) if both breakpoints fell within a repeat region, (2) if any of the breakpoints were located within <1 kb of a known assembly gap region within the reference genome, (3) mean read depth of at least 10 reads across the event. The remaining set of somatic SVs was annotated with gene information.

### Quantitative Real-time Polymerase Chain Reaction (qRT-PCR)

cDNA was synthesized using the High-Capacity cDNA Reverse Transcription Kits (Applied Biosystems, Foster City, CA) following manufacturer’s instructions. Real-time PCR was performed using the Bio-Rad iQ5 detection system with SYBR Green mix (Bio-Rad Laboratories, Hercules, CA, USA). Primer pairs used were designed with the Primer 3 RT-PCR program and checked for sequence homology against known genes using the BLAST search program^35^. Primer sequences used in this study are shown in Table 1. Calculation of the threshold value, standard curve preparation and quantification of mRNA in the samples were performed using the iQ5 software provided by Bio-Rad.

### Flow Cytometry

Flow cytometry analysis was performed using the Cytomics FC500 flow cytometer (Beckman Coulter, Fullerton, CA) as described in our previous study^36^. CSF2RA (GM-CSF receptor α chain), CRLF2 (Cytokine receptor-like factor 2), and IL3RA (IL-3 receptor α chain) levels on blood leukocytes were evaluated by flow cytometry using anti-human CD116 (Beckman Coulter, Brea, CA, USA), anti-human TSLP receptor APC, and anti-human CD123 PE-Cyanine7 (eBioscience, San Diego, CA, USA) specific antibodies respectively.

### STAT5 Phosphorylation Assay

Phosphorylation of STAT5 in blood leukocytes was evaluated by stimulation with recombinant human GM-CSF, TSLP and IL-3 (R&D Systems; Minneapolis, USA). Briefly, isolated PMBCs were incubated with GM-CSF, TSLP and IL-3 separately (10 ng/ml, 15 minutes, 37 °C). PBMCs incubated with PBS without stimulant was used as negative control individually. After the incubation, cell lysates were prepared and evaluated by Western blotting using primary antibodies against STAT5 (Santa Cruz Biotechnology, Santa Cruz, CA) and phospho-STAT5 (Millipore, Billerica, MA). Anti-β-actin antibody (Santa Cruz Biotechnology, Santa Cruz, CA) was used as a protein loading control.

### IL-1β Stimulation Experiments

PBMCs were isolated from heparinized peripheral blood using Ficoll-based density gradient centrifugation. A cell density of 10^6^ cells/ml were seeded and then stimulated with 10 ng/ml of recombinant human IL-1β (R&D Systems). Post IL-1β stimulation at time point 0, plates were incubated at 37 °C with 5% CO_2_ and cells were collected at 2 hrs. RNA was then extracted from these cells for RNA sequencing (RNA-Seq) studies. A set of baseline experiments with no stimulus was completed alongside each stimulation experiment.

### RNA Sequencing

Total RNA of PBMCs was extracted using TRIzol reagent according to manufacturer’s directions. RNA was further purified using RNeasy MiniElute Cleanup kit including a DNase digest according to the manufacturer’s instructions (QIAGEN, Valencia, CA). RNA quality and quantity were assessed using an Agilent 2100 Bioanalyzer (Agilent Technologies, Inc., Palo Alto, CA). cDNA libraries were prepared for each sample using the Illumina TruSeq RNA Sample Preparation Kit by following the manufacture’s recommended procedures. Libraries were sequenced using an Illumina HiSeq 2000 instrument with paired-end reads of 100 bp (Novogene). Approximately 3 gigabases of cDNA sequence per sample were generated.

### Analysis of Differentially Expressed Genes

Sequenced reads were aligned onto the human genome assembly hg19 using Bowtie2 in TopHat2 (v.2.0.3)^37^. Transcript assembly, abundance estimates and differential expression analyses were conducted by using Cufflinks2 (v2.2.1) and Cuffdiff2 (v2.2.1)^37^,^38^. Biological sample gene variance was not determined since differential gene expression comparison was run without biological replicates. The gene or exon level expression was normalized to the number of reads per kilobase per million mapped reads (RPKM)^39^. Genes with a *P* value smaller than 10^−2^ and a fold change greater than 2 were considered significant.

Significant genes were input into The Database for Annotation, Visualization and Integrated Discovery (DAVID) for Gene Ontology (GO) term enrichment analysis and Kyoto Encyclopedia of Genes and Genomes (KEGG) pathway analysis (http://david.abcc.ncifcrf.gov). A hypergeometric test using the Benjamini and Hochberg procedure was performed to identify significant biological pathways. Biological processes with a corrected *P* < 0.05 were considered significant.

## Additional Information

**How to cite this article**: Chiu, C.-Y. *et al*. Whole-Genome Sequencing of a Family with Hereditary Pulmonary Alveolar Proteinosis Identifies a Rare Structural Variant Involving *CSF2RA/CRLF2/IL3RA* Gene Disruption. *Sci. Rep.* **7**, 43469; doi: 10.1038/srep43469 (2017).

**Publisher's note:** Springer Nature remains neutral with regard to jurisdictional claims in published maps and institutional affiliations.

## Supplementary Material

Supplementary Information

## Figures and Tables

**Table 1:** Primer sequences for target and housekeeping genes.

Gene	Primer sequence	PCR product size
*GAPDH*	Forward: 5′-TTCCAGGAGCGAGATCCCT-3′ Reverse: 5′-CACCCATGACGAACATGGG-3′	175 bp
*CSF2RA*	Forward: 5′-TGTCGCTCTTCCCTTCTCTC-3′ Reverse: 5′-TTCGCAGATCCGATTTCTCT-3′	116 bp
*CRLF2*	Forward: 5′-TGCACCAACTACCTTCTCCA-3′ Reverse: 5′-TGATGCCACGAAAATCTCAC-3′	182 bp
*IL3RA*	Forward: 5′-TTAAGCAGGCACCTCTGTCC-3′ Reverse: 5′-CTGAGCCTTTGCTTTCATCC-3′	154 bp

PCR, polymerase chain reaction; *GAPDH*, glyceraldehyde 3-phosphate dehydrogenase; *CSF2RA*, colony stimulating factor 2 receptor, alpha; *CRLF2*, cytokine receptor-like factor 2; *IL3RA*, interleukin 3 receptor, alpha.
