# Supplementary material for: Whole-Genome Sequencing of a Family with Hereditary Pulmonary Alveolar Proteinosis Identifies a Rare Structural Variant Involving CSF2RA/CRLF2/IL3RA Gene Disruption
Source: Sci Rep. 2017 Feb 24;7:43469. doi: 10.1038/srep43469 (PMC5324064; doi:10.1038/srep43469)
Supplement: Supplementary Information [file srep43469-s1.pdf]

Supplementary information

Whole-Genome Sequencing of a Family with Hereditary Pulmonary Alveolar Proteinosis Identifies a Rare Structural Variant Involving *CSF2RA/CRLF2/IL3RA* Gene Disruption

Chih-Yung Chiu, Shih-Chi Su, Wen-Lang Fan, Shen-Hao Lai, Ming-Han Tsai, Shih-Hsiang Chen, Kin-Sun Wong & Wen-Hung Chung

Correspondence and requests for materials should be addressed to
K.-S.W. (email: pchest@adm.cgmh.org.tw) or W.-H.C. (email: chung1@cgmh.org.tw)

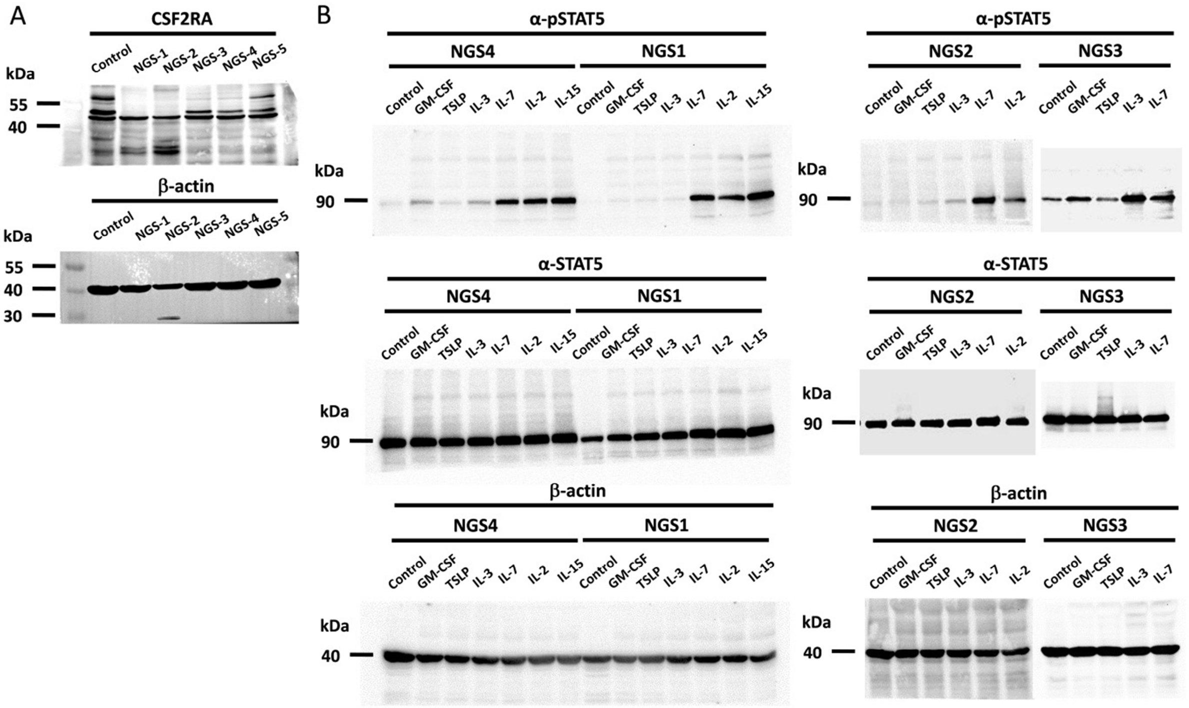

Supplementary Figure S1. Full-length blots/gels for Figure 1E (A) and Figure 3C (B).

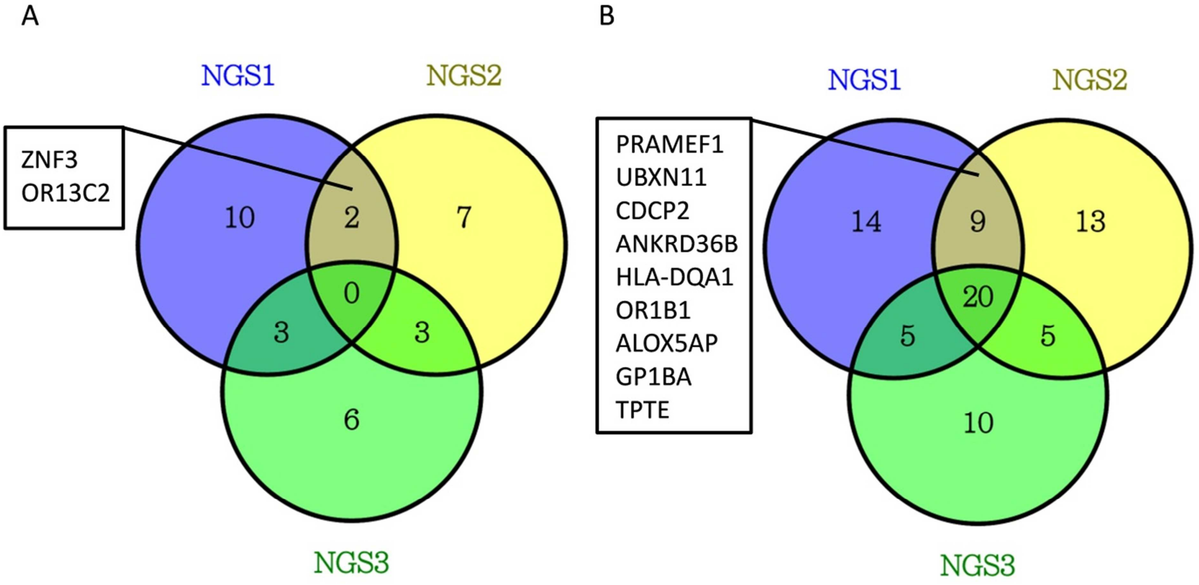

Supplementary Figure S2. Venn diagrams indicate the number of filtered candidates at homozygous (A) and compound heterozygous (B) states. To identify autosomal or sex-linked recessive mutations that inactivate (truncate) the gene product, we considered ANNOVAR-annotated variants with the following criteria: (1) loss-of-function: splicing, stop-loss/-gain, and frameshift variants (variant calls in tandem repeat regions obtained from the UCSC Genome Browser were excluded); (2) minor allele frequency (MAF) < 1%: The MAF annotations used included data from two different populations of the 1000 Genomes Project and the ExAC dataset (EAS and SAS), and a panel of 267 germline DNA samples (Chang Gung Human Database, an unpublished whole-genome database of normal controls); (3) exclusion of potential false-positive calls: we visualized the sequencing reads surrounding the remaining variants using the Integrative Genomics Viewer (IGV) to improve the accuracy of variant filtering since detection of variants within segmental duplication region is more challenging. After filtering, homozygous mutations of 48 and 47 genes were identified in the proband and his asymptomatic brother, respectively. Among these candidates, 2 genes were detected in both the proband and his asymptomatic brother but not in the normal sister. Moreover, compound heterozygous mutations of 48 and 47 genes were recovered in the proband and his asymptomatic brother, respectively, with 9 genes being shared in both the proband and his asymptomatic brother but not in the normal sister. Although these genes appeared to be not directly related to PAP, *GP1BA* gene has been functionally implicated in bacterial clearance and protecting hosts from pathogen infections (Wong et al, Nat Immunol 2013; 14: 785-92), while *ALOX5AP* gene polymorphisms have been shown to be associated with asthma and allergy (Holloway et al, Allergy 2008; 63:1046-53), which may partially explain the differential manifestation of PAP in the family.

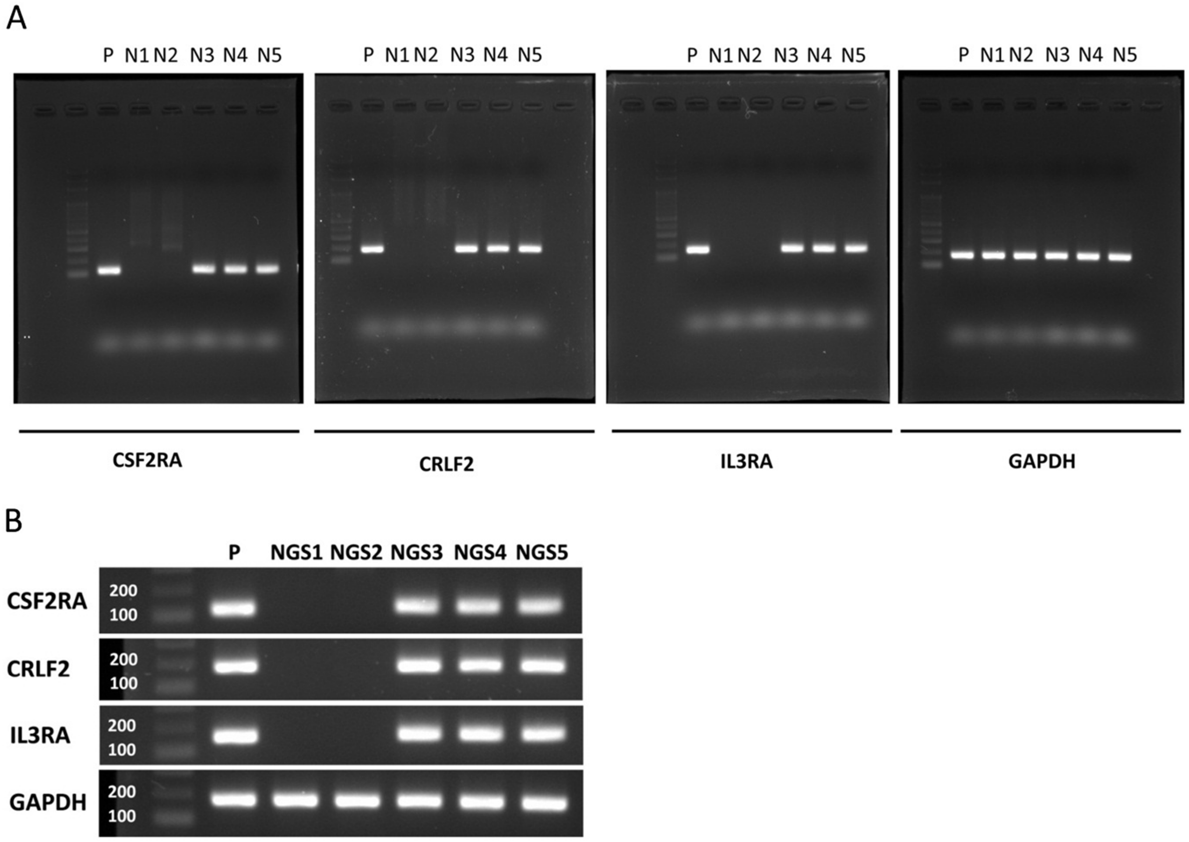

Supplementary Figure S3. Full-length blots/gels for RT-PCR (A). Agarose gel electrophoresis revealed the absence of PCR products obtained from *CSF2RA*, *CRLF2*, and *IL3RA* genes in NGS1 and NGS2 (B).

Supplementary Table S1. Statistics of variant filtering.

Autosomal recessive model				
**Homozygous mutation**				
	NGS1	NGS2	NGS3	(NGS1∩NGS2)-NGS3
SNP (Variant Level)	178,344	168,081	165,584	
Indel (Variant Level)	27,826	26,420	26,324	
Filtered SNP (Variant Level)	9	4	3	0
Filtered Indel (Variant Level)	7	12	13	0
Filtered SNP+Indel (Gene Level)	15	12	12	2
**Compound heterozygous mutation**				
	NGS1	NGS2	NGS3	(NGS1∩NGS2)-NGS3
SNP (Variant Level)	1,910,287	1,949,575	1,983,453	
Indel (Variant Level)	298,055	315,890	319,807	
Filtered SNP (Variant Level)	119	135	130	27
Filtered Indel (Variant Level)	203	206	205	37
Filtered SNP+Indel (Gene Level)	48	47	40	9

Filter conditions included stopgain, stoploss, frameshift and splicing. Abbreviations: SNP, single nucleotide polymorphism; Indel, insertion and deletion.

Supplementary Table S2. KEGG and Enrichment of GO BP annotations for 21 genes identified in all three subjects and 63 genes identified just in NGS2 and NGS3 rather than NGS1.

Category	Term	Count	%	P Value	Genes	List Total	Pop Hits	Pop Total	Fold Enrichment	FDR
**21 genes**										
**Annotation Cluster 1**	**Enrichment Score: 3.975**									
GOTERM_BP_FAT	GO:0006935~chemotaxis	7	4.375	1.81E-08	CXCL1, CCL20, CXCL2, CCR2, FPR3, CXCR2, PLAU	17	160	13528	34.815	2.59E-05
GOTERM_BP_FAT	GO:0042330~taxis	7	4.375	1.81E-08	CXCL1, CCL20, CXCL2, CCR2, FPR3, CXCR2, PLAU	17	160	13528	34.815	2.59E-05
GOTERM_BP_FAT	GO:0006954~inflammatory response	8	5.000	4.11E-08	CXCL1, NFKBIZ, CCL20, HMOX1, CXCL2, CCR2, CXCR2, IL1A	17	325	13528	19.588	5.90E-05
GOTERM_BP_FAT	GO:0009611~response to wounding	9	5.625	5.14E-08	CXCL1, NFKBIZ, CCL20, HMOX1, CXCL2, CCR2, CXCR2, PLAU, IL1A	17	530	13528	13.513	7.36E-05
GOTERM_BP_FAT	GO:0007626~locomotory behavior	7	4.375	4.41E-07	CXCL1, CCL20, CXCL2, CCR2, FPR3, CXCR2, PLAU	17	274	13528	20.330	6.33E-04
GOTERM_BP_FAT	GO:0007610~behavior	8	5.000	5.02E-07	CXCL1, PTGS2, CCL20, CXCL2, CCR2, FPR3, CXCR2, PLAU	17	469	13528	13.574	7.20E-04
GOTERM_BP_FAT	GO:0006952~defense response	8	5.000	3.10E-06	CXCL1, NFKBIZ, CCL20, HMOX1, CXCL2, CCR2, CXCR2, IL1A	17	615	13528	10.351	0.004
KEGG_PATHWAY	hsa04060:Cytokine-cytokine receptor interaction	7	4.375	6.56E-06	CXCL1, TNFRSF10C, CCL20, CXCL2, CCR2, CXCR2, IL1A	12	262	5085	11.322	0.005
**63 genes**										
**Annotation Cluster 1**	**Enrichment Score: 6.678**									
GOTERM_BP_FAT	GO:0009611~response to wounding	22	36.667	2.83E-16	IRAK2, IL6, CCL3, TNF, PLEK, ADORA2A, CXCL3, IL1RN, CXCR1, CCL4L2, NLRP3, CCL4, SOD2, CD163, ORM1, TNFAIP6, EREG, ADM, F3, IL1B, ORM2, PTAFR	55	530	13528	10.210	5.440E-13
GOTERM_BP_FAT	GO:0006954~inflammatory response	18	30.000	4.14E-15	IRAK2, CCL3, IL6, TNF, ADORA2A, CXCL3, IL1RN, CXCR1, CCL4L2, NLRP3, CCL4, CD163, ORM1, TNFAIP6, F3, IL1B, ORM2, PTAFR	55	325	13528	13.623	6.650E-12
GOTERM_BP_FAT	GO:0006952~defense response	19	31.667	1.13E-11	IRAK2, IL6, CCL3, TNF, ADORA2A, NCF1, CXCL3, IL1RN, CXCR1, CCL4L2, NLRP3, CCL4, CD163, ORM1, TNFAIP6, F3, IL1B, ORM2, PTAFR	55	615	13528	7.599	1.823E-08
GOTERM_BP_FAT	GO:0006955~immune response	18	30.000	7.05E-10	ICAM1, IL1R2, IL6, CCL3, NBN, TNF, AQP9, NCF1, CXCL3, IL1RN, TNFSF15, CCL4L2, NLRP3, CCL4, CLEC4E, CD274, IL1B, PTAFR	55	690	13528	6.416	1.140E-06

GOTERM_BP_FAT	GO:0007626~locomotory behavior	10	16.667	1.20E-06	CCL3, IL6, ADORA2A, CXCL3, CXCR1, IL1B, CCL4L2, CCL4, PTAFR, SOD2	55	274	13528	8.977	0.002
GOTERM_BP_FAT	GO:0006935~chemotaxis	8	13.333	3.15E-06	CCL3, IL6, CXCL3, CXCR1, IL1B, CCL4L2, CCL4, PTAFR	55	160	13528	12.298	0.005
GOTERM_BP_FAT	GO:0042330~taxis	8	13.333	3.15E-06	CCL3, IL6, CXCL3, CXCR1, IL1B, CCL4L2, CCL4, PTAFR	55	160	13528	12.298	0.005
KEGG_PATHWAY	hsa04060:Cytokine-cytokine receptor interaction	10	16.667	1.65E-05	IL1R2, CCL3, IL6, TNF, CXCL3, TNFSF15, CXCR1, IL1B, CCL4L2, CCL4	32	262	5085	6.065	0.017
**Annotation Cluster 2**	**Enrichment Score: 2.576**									
GOTERM_BP_FAT	GO:0002237~response to molecule of bacterial origin	7	11.667	1.12E-06	IL6, ADM, SOCS3, NFKBIA, IL1B, IRG1, PTAFR	55	86	13528	20.020	0.002
GOTERM_BP_FAT	GO:0009617~response to bacterium	8	13.333	1.08E-05	IL6, TNF, ADM, SOCS3, NFKBIA, IL1B, IRG1, PTAFR	55	193	13528	10.195	0.018
GOTERM_BP_FAT	GO:0032496~response to lipopolysaccharide	6	10.000	1.33E-05	ADM, SOCS3, NFKBIA, IL1B, IRG1, PTAFR	55	77	13528	19.166	0.022

Pathways were identified by DAVID Functional Annotation and selected by using a FDR adjusted *P* value < 0.05. Abbreviations: KEGG, Kyoto Encyclopedia of Genes and Genomes; GO, Gene Ontology; BP, biological process.
